# Exploring COVID-19 Pandemic Disparities with Transcriptomic Meta-analysis from the Perspective of Personalized Medicine

**DOI:** 10.1007/s12275-024-00154-9

**Published:** 2024-07-09

**Authors:** Medi Kori, Ceyda Kasavi, Kazim Yalcin Arga

**Affiliations:** 1 Institute of Health Sciences, Acibadem Mehmet Ali Aydinlar University, 34752 Istanbul, Turkey; 2 Faculty of Health Sciences, Acibadem Mehmet Ali Aydinlar University, 34752 Istanbul, Turkey; 3 Department of Bioengineering, Marmara University, 34722 Istanbul, Turkey; 4 Genetic and Metabolic Diseases Research and Investigation Center, Marmara University, 34722 Istanbul, Turkey

**Keywords:** COVID-19, Bioinformatics, Transcriptome, Gender and age disparities, Personalized medicine, Host-directed drug candidates

## Abstract

Infection with SARS-CoV2, which is responsible for COVID-19, can lead to differences in disease development, severity and mortality rates depending on gender, age or the presence of certain diseases. Considering that existing studies ignore these differences, this study aims to uncover potential differences attributable to gender, age and source of sampling as well as viral load using bioinformatics and multi-omics approaches. Differential gene expression analyses were used to analyse the phenotypic differences between SARS-CoV-2 patients and controls at the mRNA level. Pathway enrichment analyses were performed at the gene set level to identify the activated pathways corresponding to the differences in the samples. Drug repurposing analysis was performed at the protein level, focusing on host-mediated drug candidates to uncover potential therapeutic differences. Significant differences (i.e. the number of differentially expressed genes and their characteristics) were observed for COVID-19 at the mRNA level depending on the sample source, gender and age of the samples. The results of the pathway enrichment show that SARS-CoV-2 can be combated more effectively in the respiratory tract than in the blood samples. Taking into account the different sample sources and their characteristics, different drug candidates were identified. Evaluating disease prediction, prevention and/or treatment strategies from a personalised perspective is crucial. In this study, we not only evaluated the differences in COVID-19 from a personalised perspective, but also provided valuable data for further experimental and clinical efforts. Our findings could shed light on potential pandemics.

**Supplementary Information:**

The online version contains supplementary material available at 10.1007/s12275-024-00154-9.

## Introduction

Coronavirus disease 2019 (COVID-19) is caused by infection with severe acute respiratory syndrome coronavirus 2 (SARS-CoV2). It was first identified in Wuhan, in December 2019 and spread rapidly worldwide through person-to-person transmission (Yuki et al., 2020). The ongoing COVID-19 pandemic caused more than six million deaths and more than seven hundred sixty million cases of illness in the world by April 2023, according to WHO statistics (WHO, 2023). SARS-CoV2 primarily affects the respiratory system, and various symptoms ranging from asymptomatic to life-threatening (i.e., acute myocardial infarction, systemic inflammation, or multiple organ failure) have been associated with the infection (Zhang et al., 2022a, 2022b). As COVID-19 has a global impact on healthcare and has various clinical manifestations, understanding the disease and presenting effective prevention and/or treatment strategies are still under discussion and subject of research.

A substantial number of population-based studies have shown that gender, age, and/or the presence of various diseases (e.g., hypertension, diabetes, and cancer) can influence COVID-19 severity. It has been reported that males and the elderly are significantly associated with poor COVID-19 outcomes (Doerre & Doblhammer, 2022; Zhang et al., 2022a, 2022b; Zhou et al., 2020). Therefore, COVID-19 also emphasizes the importance of patient-to-patient differences in the development or treatment of any disease. Moreover, the "one size fits all" paradigm is not efficient for many diseases, including COVID-19, which leads us to the concept of "personalized medicine".

The presence of multifactorial effects, the current lack of robust diagnostic and/or prognostic biomarkers, and the morbidity and mortality risk associated with COVID-19 in vulnerable populations call for novel approaches to identify underlying disease mechanisms, biomarker candidates, drug targets, or drug candidates. Considering these needs, researchers have conducted many bioinformatics approaches to find solutions to these needs (Auwul et al., 2021; Fang et al., 2021; Hoque et al., 2022; Sameh et al., 2023). However, these studies are insufficient when it comes to a personalized perspective. The major limitation of these previous studies is that they did not consider the gender or/and age of the patients and did not perform a meta-analysis that considers these differences.

In the current study, considering the possible differences in gene expression profiles according to the collection source, gender, age of patients, and viral load of patients we performed a meta-analysis of transcriptome datasets between COVID-19 and control phenotypes to emphasize the personalized view in COVID-19 and to present differences at the transcript, pathway, and drug levels. Accordingly, in this study, a meta-analysis of the eight gene expression datasets associated with COVID-19 was performed and common differentially expressed genes (core-DEGs) between cases were identified. Because host proteins are recognized as effective drug candidates for infectious diseases, we integrated our core-DEGs with the SARS-CoV-2 and human network for each case to identify potential drug targets. Finally, we performed drug repurposing analysis for each case to find host-targeted drugs. Consequently, with this study, we believe we have highlighted the importance of personalized approaches by revealing the differences between the disease and control phenotypes at multiple levels, and we have also presented host-mediated, powerful therapeutic biomarkers for further experimental and clinical studies for COVID-19.

## Materials and Methods

### Data Extraction: Gene Expression Datasets

For the meta-analysis of COVID-19 gene expression profiles, the Gene Expression Omnibus (GEO) database repository (Barrett et al., 2013) was systematically searched. The following criteria were used to select datasets: (i) samples should consist of COVID-19 and control phenotypes, (ii) COVID-19 status of participants should be validated by RT-PCR or imaging (i.e. X-ray or chest computed tomography), (iii) gender and age of participants should be reported, (iv) samples should be originated from human sources, and (v) the same sequencing strategy and platform should be used (i.e. Illumina).

### Sample Classification for Study Design

**Fig. 1:**
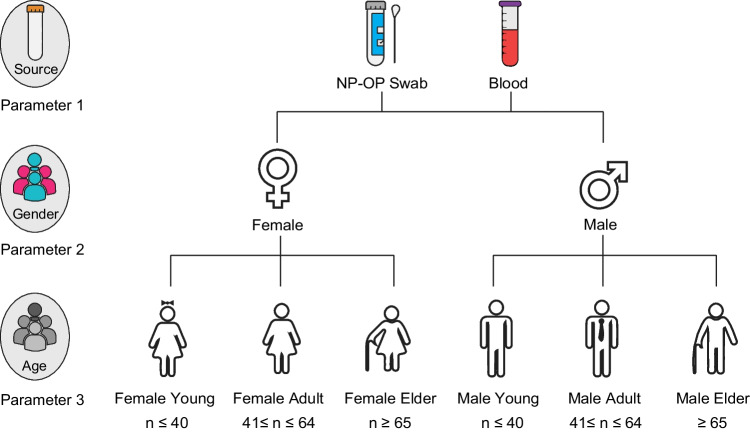
The parameters considered in the meta-analysis of gene expression datasets. In the analysis, samples were classified according to the collection source (nasopharyngeal and oropharyngeal (NP / OP) swabs or blood) (Parameter 1), gender (Parameter 2), and age (Parameter 3)

Samples from the gene expression datasets used in this study were grouped according to three parameters, including source (Parameter 1), gender (Parameter 2) and age (Parameter 3) (Fig. 1).

Being aware of the possible differences in gene expression profiles between the different source types, we first categorised the datasets into two groups according to their origin: respiratory tract (nasopharyngeal and oropharyngeal [NP / OP] swabs) and blood.

Given the strong gender differences in risk for COVID-19 (Kharroubi & Diab-El-Harake, 2022), the samples were then classified as female and male based on gender.

Moreover, considering the significant influence of age on mortality in COVID-19 patients (Bonanad et al., 2020), the samples were divided into three categories: young, adult and elderly to define age-related differences. It has been reported that individuals younger than 40 years old are less likely to be infected with COVID-19 and, if infected, have a lower risk of death (Zhang et al., 2022a, 2022b). As mortality increases with age, the Centres for Disease Control and Prevention (CDC) recommends that people aged 65 and older should be vaccinated (CDC, 2023). Accordingly, samples were defined as "young" if they were younger than or equal to 40 years of age (n ≤ 40), they were defined as "adult" if they were between 41 and 64 years of age (41 ≤ n ≤ 64), and they were defined as "elder" if they were older than or equal to 65 years of age (n ≥ 65).

Given the conflicting associations in the literature between (i) COVID‐19 disease severity and viral load and (ii) viral load and patient age (Abdulrahman et al., 2021; Acer et al., 2022; Dadras et al., 2022), we categorised COVID-19 patients into high and low viral load to understand the possible relationship between viral load, gender and age. In the study, the patients' cycle threshold (CT) and viral reads-per-million (rpM) values were taken into account to categorise patients into a low or high group. A low Ct value corresponds to a high viral load and in the study, patients with a CT ≤ 24 considered as high viral load (i.e. CT > 24 as low viral load) and the samples in the SARS-CoV-2 groups with at least viruses with values > 1,000 rpM are considered to have a high viral load (i.e. ≤ 1,000 rpM as a low viral load) (Platten et al., 2021).

### Identification of Differentially Expressed Genes

Each dataset of gene expression data from COVID-19 patients and healthy controls was analysed individually to identify DEGs. If one of the phenotypes (i.e. COVID-19 and controls) contained less than 3 samples, no DEG analyses were performed for this case considering the design parameters.

The DESeq2 package (Love et al., 2014) in R/Bioconductor (version 4.0.2) (Huber et al., 2015) was used to identify DEGs for each case. DESeq2 fits a generalized linear model of the negative binomial family and uses the Wald test for statistical significance. The Benjamini–Hochberg method was used to control for false discovery rate, and an adjusted p-value threshold of 0.05 was used to determine statistical significance. Fold change was used to determine the regulatory patterns of DEGs, and at least a twofold change was considered significant.

The DEGs of the individual datasets were comparatively analysed to identify common signatures of COVID-19, and further analyses were performed using the common DEGs, referred to as "core-DEGs". Due to sample size limitation, the comparative analysis cannot be performed in all cases, and the core-DEGs were defined as common DEGs of at least "n-1" records when "n > 2". For example, if four datasets could be used for DEG analyses, common DEGs in at least three datasets were accepted as "core-DEGs ". If two datasets could be used, common DEGs in both datasets were accepted as "core-DEGs", and analyses restricted to a single dataset were excluded from further analyses.

### Multivariate Pathway Enrichment Analysis

To gain biological insights into the culminated relevant case-core-DEGs, an integrative statistical method called "ActivePathways" (Paczkowska et al., 2020) was implemented using the R platform (version 4.0.2) (Huber et al., 2015). For the analysis, the gene sets corresponding to the molecular pathways in the Reactome database were downloaded from the Molecular Signatures Database (MSigDB) (Liberzon et al., 2015). Pathway enrichment analysis was performed independently for each case. During the analysis, the input list (i.e., the relevant case-core-DEGs) was combined with their adjusted p-values using Fisher's method. The Benjamini–Hochberg method was used to control for FDR. The adjusted *p*-value cut-off of 0.01 was used to determine the statistical significance of the molecular pathways. To avoid statistical bias, significant pathways associated with fewer than three core-DEGs were excluded.

### Reconstruction of SARS-CoV-2 and Human Protein–Protein Interaction Networks

The protein–protein interactions (PPIs) between SARS-CoV-2 and humans were extracted from the BioGRID database (v.4.4.217) (Oughtred et al., 2021). The networks of SARS-CoV-2 and the human PPIs were reconstructed for each case using the relevant case core-proteins. The reconstructed networks were represented as undirected graphs, with the nodes representing the proteins and the edges representing the interactions between the virus and the human proteins. The graphs were visualized using Cytoscape (v.3.7.0) (Shannon et al., 2003).

### Drug Repurposing Analysis

PharmOmics, a drug repositioning and toxicity prediction platform, has been used to reveal drug signatures of core-proteins (Chen et al., 2022). Since drug therapies that specifically target host proteins offer great potential for the present and future, especially in infectious diseases (Kori et al., 2023), we used core-host proteins (i.e. proteins that interact with viral proteins) for drug repositioning analysis. To obtain more robust results, we used core-proteins as input if their regulatory patterns (i.e. up- or down-regulation) at the mRNA level were consistent in the datasets. We considered results from in vitro studies and only retrieved results from the L1000 database. For gene overlap-based drug repositioning, the platform measures direct gene overlap with the drug and uses Fisher’s exact test to obtain p-values. The 10 drugs with the highest p-value were considered as potential drug candidates in this study. The drug candidate descriptions are taken from the L1000 Fireworks Display (L1000FWD) application (Wang et al., 2018).

## Results

### Gene Expression Datasets of COVID-19

A total of eight gene expression datasets with accession numbers GSE152075 (Lieberman et al., 2020), GSE152641 (Thair et al., 2020), GSE156063 (Mick et al., 2020), GSE157103 (Overmyer et al., 2021), GSE161731 (McClain et al., 2021), GSE167000 (Galbraith et al., 2022), GSE179277 (Mick et al., 2021) and GSE188678 (unpublished) met the inclusion criteria and were selected for analysis. The samples from datasets GSE179277, GSE188678, GSE152075, and GSE156063 were from the respiratory tract, while the samples from GSE152641, GSE157103, GSE161731, and GSE167000 were from the blood.

**Fig. 2:**
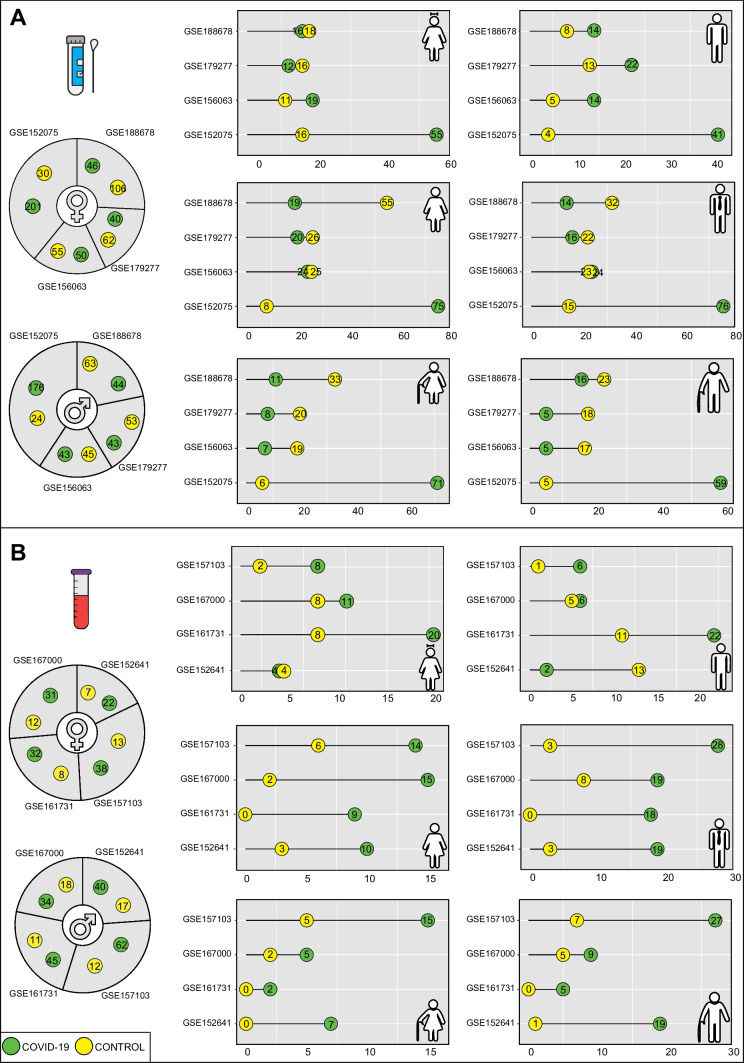
The number of samples used in the meta-analysis of the transcriptome datasets. **A** The number of samples corresponding to the source of nasopharyngeal and oropharyngeal swab samples. **B** The number of samples corresponding to the source of blood collection. The pie charts show the number of samples considering gender differences and the dot charts show the gender and age differences between COVID-19 and control phenotypes. The green color corresponds to the COVID-19 patient phenotype, while the yellow corresponds to the control phenotype

Taking into account the defined parameters, a total of 1081 respiratory tract samples and 402 blood samples were used for the analyses. Of the respiratory samples, 337 females and 306 males have COVID-19, while 253 females and 185 males are controls. Of the female cases, 163 are young (102 cases COVID-19 positive, including 68 with high viral load, 31 with low viral load and 3 unknown), 252 are adults (138 cases COVID-19 positive, including 92 with high viral load, 45 with low viral load and 1 unknown) and 175 (97 cases COVID-19 positive, including 66 with high viral load, 28 with low viral load and 3 unknown) are elder. Of the male cases, 121 are young (91 COVID-19 positive, including 57 with high viral load, 32 with low viral load and 2 unknown), 222 are adults (130 COVID-19 positive, including 73 with high viral load, 55 with low viral load and 2 unknown) and 148 are elder (85 COVID-19 positive, including 61 with high viral load, 18 with low viral load and 6 unknown) (Fig. 2A, Table S1).

Of the blood samples, 163 are female (123 cases COVID-19 positive) and 239 are male (181 cases COVID-19 positive). When the female and male samples are classified by age, 65 females (43 cases COVID-19 positive) and 66 males (36 cases COVID-19 positive) are young, 59 females (48 cases COVID-19 positive) and 98 males (84 cases COVID-19 positive) are adult, 36 females (29 cases COVID-19 positive) and 73 males (60 cases COVID-19 positive) are elder (Fig. 2B). Since these expression data belonging to the blood source do not contain clinical information on viral load, the viral load effect for these datasets cannot be analysed.

### Differentially Expressed Genes in COVID-19

**Fig. 3:**
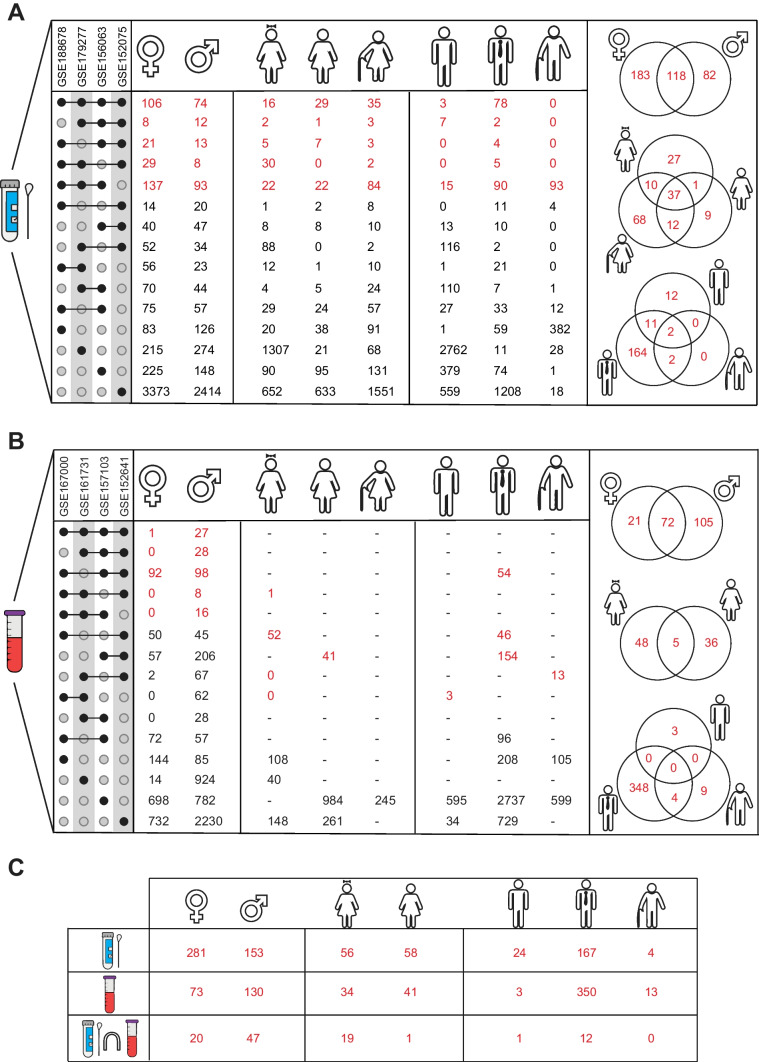
The number of differentially expressed genes (DEGs) in the different cases. **A** The number of DEGs found for the nasopharyngeal and oropharyngeal swab sources. Venn diagrams represent the common DEG number between cases. Red numbers represent core-DEG numbers accepted in the study. **B** The number DEGs found for blood sources. Venn diagrams show the common DEG number between cases. The red numbers represent the core-DEG numbers that were accepted in the study. **C** The results of the comparative analysis of the core-DEGs

DEG analyses were performed for each case in four respiratory source datasets (GSE179277, GSE188678, GSE152075 and GSE156063), and DEGs identified in at least three of the four datasets were accepted as "core-DEGs". The analyses yielded 301 and 200 core-DEGs for female and male cases, respectively. In total, 75 core-DEGs were identified for the young female, 59 core-DEGs for the adult female, 127 core-DEGs for the elder female, 25 core-DEGs for the young male, 179 core-DEGs for the adult male, and 93 core-DEGs for the elder male. In addition, taking into account differences in viral load (i.e. high or low viral load), 10 core- DEGs were identified for female cases, 3 core-DEGs for male cases and one core-DEG each for adult female (*SLITRK2*), young male (*RUNX1T1*) and older male (*GRIA4*) cases (Table S1). Comparative analysis of the core-DEGs showed that 118 core-DEGs were observed in both the female and male cases combined. Furthermore, the comparison of the core-DEGs considering the age parameter (i.e. young, adult and elder) revealed 37 common core-DEGs in the female case, while only 2 common core-DEGs were identified in the male case (Fig. 3A).

Due to the 3-sample restriction, the DEG analysis cannot be performed for all four blood source datasets (GSE152641, GSE157103, GSE161731, and GSE167000). Accordingly, considering the sample restriction criteria that defined for the core-DEG assumption, we obtained a total of 93 core-DEGs for the female, 177 core-DEGs for the male, 53 core-DEGs for the young female, 53 core-DEGs for the adult female, 3 core-DEGs for the young male, 254 core-DEGs for the adult male, and 13 core-DEGs for the elder male cases. A comparative analysis of these core- DEGs revealed 72 common core-DEGs between female and male cases. Furthermore, when we compared the common core-DEGs between female cases taking age differences into account, we obtained 5 common core-DEGs, whereas we obtained no common core-DEGs in male cases taking age differences into account (Fig. 3B).

To further investigate the differences and similarities between sources, gender, and age in the COVID-19 phenotype, we comparatively analysed the resulting core-DEGs in all cases (Fig. 3C).

### Molecular Pathways Involved in COVID-19

**Fig. 4:**
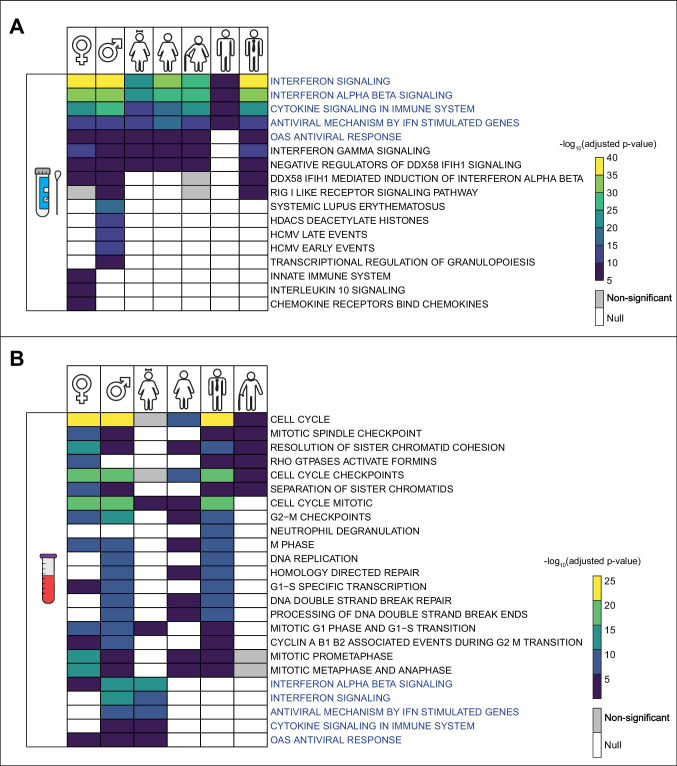
Pathway enrichment analysis results of the core-DEGs. **A** Core-DEGs belonging to the source of nasopharyngeal and oropharyngeal swab specimens. **B** Core-DEGs belonging to the source of the blood samples. Pathways highlighted in blue represent common pathways for both sources

It is known that infections can alter molecular signaling pathways to their advantage. Therefore, to gain insight into the biological background of the disease, we performed an analysis of pathway enrichment, taking into account differences in origin, gender and age of the cases. Since we could not obtain a sufficient number of core-DEGs (n ≤ 10) associated with viral load, we could not perform pathway enrichment analyses considering the viral load parameter. The core-DEGs of the respiratory samples were associated with signaling pathways, including interferon signaling pathways, cytokine signaling pathways, DDX58 IFIH1 signaling pathways, RIG-I-like receptor signaling pathways, and interleukin-10 signaling pathways (Fig. 4A). No significant differences were found in the highlighted signaling pathways depending on the age of the female samples. In general, we found several specific signaling pathways in males (e.g., the RIG-I-like receptor signaling pathway). We found no significant signaling pathway (adjusted *p*-value < 0.01) associated with the elder male case.

On the contrary, the core-DEGs of blood source were associated with cell cycle, DNA replication, pathways related to cell cycle checkpoints and cell cycle phases (Fig. 4B). We did not find any significant pathways (adjusted *p*-value < 0.01) associated with the elder female and young male cases.

The primary site of SARS-CoV-2 infection is the respiratory tract, and the RNA of this virus has been detected in blood samples, especially in patients with mild symptoms. Therefore, SARS-CoV-2 infection can trigger immune responses in both the respiratory tract and the blood. Interferons are a family of cytokines that play an important role in the immune response to viruses, including SARS-CoV-2 (Lopez et al., 2021). RIG-I, which is encoded by the *DDX58* gene in the human genome, provides an immune response to most viruses that potentially contain an RNA genome, including coronaviruses, which are RNA viruses (Brisse & Ly, 2019). In addition, interleukin-10, like interferons, is a cytokine whose overexpression is associated with inflammation and immunity as well as with the progression of COVID-19 (Lu et al., 2021).

### Reconstruction of SARS-CoV-2- Human Protein Interaction Networks to Reveal Host Proteins

**Fig. 5:**
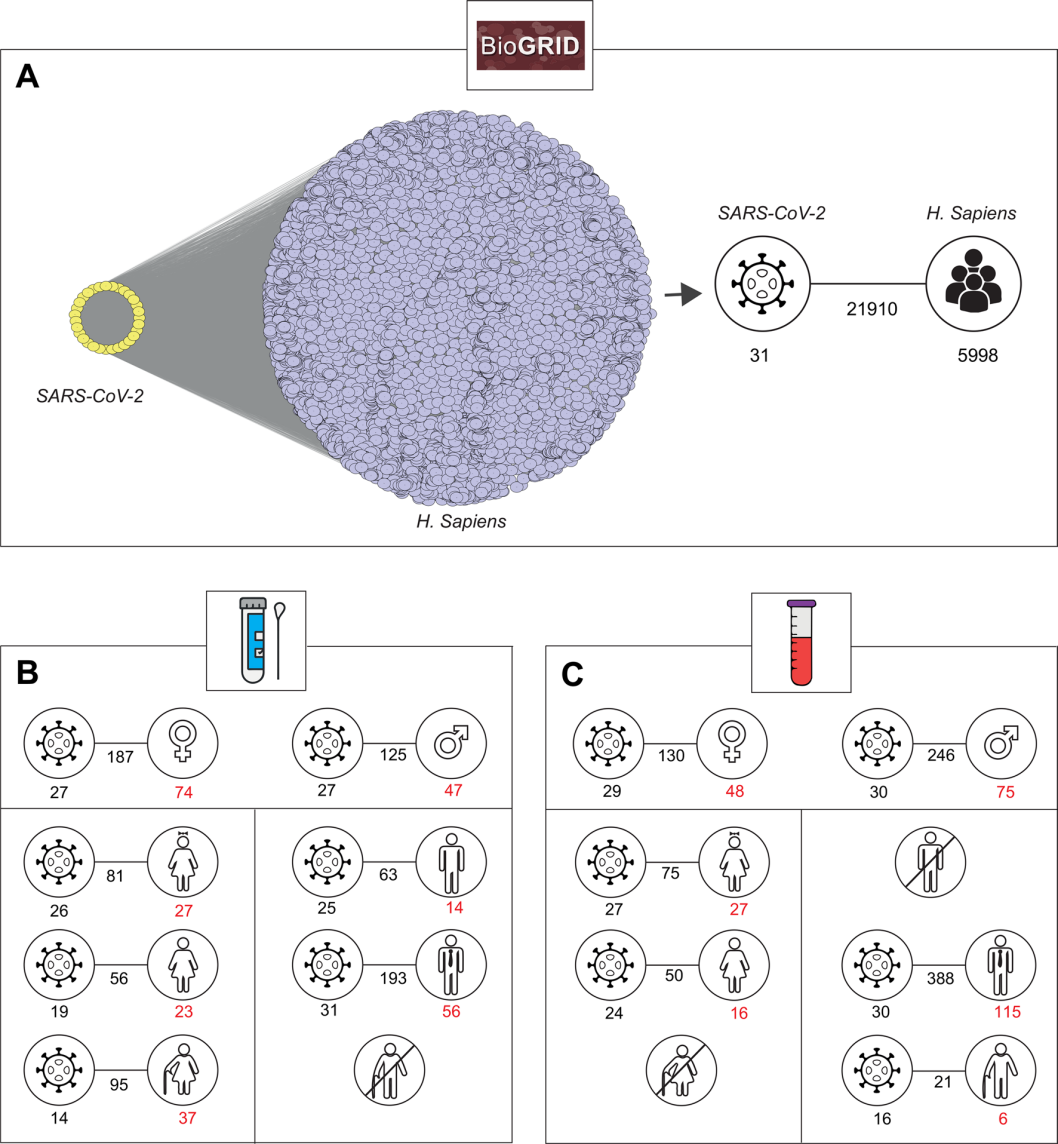
Reconstruction of SARS-CoV-2 and human protein interaction networks. **A** Protein–protein interaction network between SARS-CoV-2 and humans obtained from database resource. **B** Integration of viral proteins with core-DEGs resulted from nasopharyngeal and oropharyngeal swab sources. **C** Integration of viral proteins with core-DEGs resulted from blood sources

PPIs between SARS-CoV-2 and humans were extracted from the BioGRID database (Oughtred et al., 2021), which contains 21,910 physical interactions between 31 viruses and 5,998 human proteins (Fig. 5A). The proteins M, ORF7b and nsp4 were the viral proteins that showed the most interactions with human proteins (8.6%, 7.3% and 6.7%, respectively). One membrane protein, M, plays an important role in virus assembly and morphogenesis, but the underlying process between the M protein and virus assembly was not clear (Zhang et al., 2022a, 2022b). Although the function of ORF7b, one of the accessory proteins of SARS-CoV-2, is not yet clear, one of the most recent studies suggests that it mediates TNF-induced apoptosis (Yang et al., 2021). Of the few reports, a recent study suggests that a non-structural protein, nsp4, induces mitochondrial DNA release (Faizan et al., 2022). The top 3 host proteins with the most interactions with viral proteins are PFKP, PGRMC1 and MYO1B. Interestingly, their possible association and/or function in COVID-19 has not yet been investigated.

When the core-DEGs from each case belonging to the respiratory sources were integrated with SARS-CoV-2 and human interactions, we obtained that 74 and 47 of the female and male core- DEGs had interactions with a total of 27 SARS-CoV-2 proteins with a number of 187 and 125 interactions, respectively. In addition, 27 of the young female core-DEGs, 23 of the adult female core-DEGs, 37 of the older female core-DEGs, 14 of the young male core-DEGs, and 56 of the adult male core-DEGs have interactions with SARS-CoV-2 proteins (Fig. 5B).

When the core-DEGs from the blood sources for each case are integrated with the SARS-CoV-2 and human interactions, we obtain that 48 and 75 of the female and male core-DEGs have interactions with a total of 29 and 30 SARS-CoV-2 proteins, respectively. In addition, 27 of the young female core-DEGs, 16 of the adult female core-DEGs, 115 of the adult male core-DEGs and 6 of the elder male core-DEGs have interactions with SARS-CoV-2 proteins (Fig. 5C).

We could not find any host proteins (i.e. DEGs encoding host proteins that interacts with SARS CoV-2 proteins) for the elder male core-DEGs from the respiratory source, and for the elder female and young male core-DEGs from the blood source, so these cases were excluded from further drug repurposing analysis.

### The Candidate Repurposed Therapeutic Agents for COVID-19

**Fig. 6:**
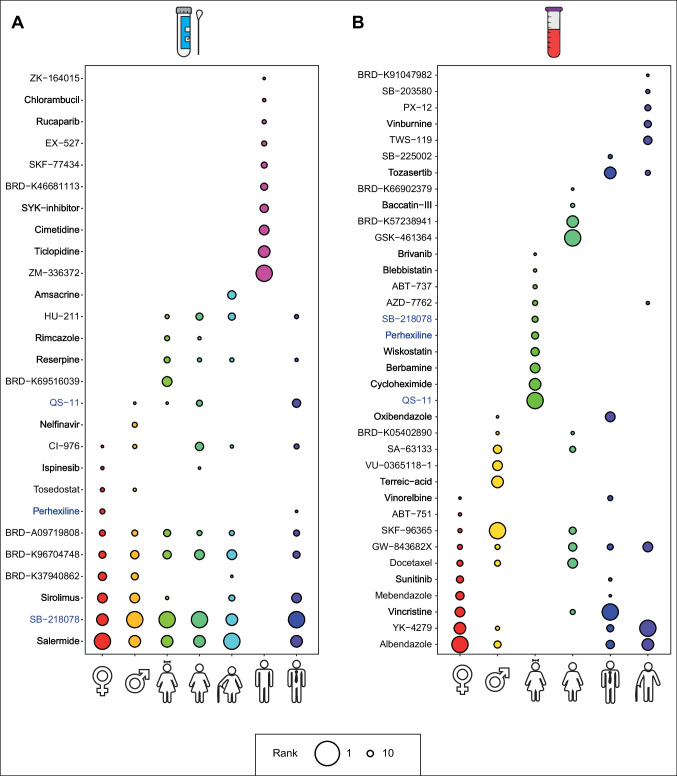
Results of the analysis of drug repurposing. **A** Top ten drug candidates resulted from nasopharyngeal and oropharyngeal swabs. **B** The top ten drug candidates resulted from blood sources. The size of the circle varies with statistical significance (*p*-value), and the most significant drug candidate is represented by the largest circle. Drugs highlighted in blue represent common drug candidates for both sources

Knowing that in infectious diseases host proteins represent efficient treatment outcomes compared to any unspecified target protein, we used DEGs encoding core-host proteins as input for the drug repositioning analysis. We considered the 10 drugs with the highest p-value and ranked the drugs according to their statistical significance. A total of 27 and 36 different drug candidates were found for respiratory tract (Fig. 6A) and blood (Fig. 6B) cases, respectively. Perhexiline, SB-218078 and QS-11 were frequently observed in both sources. In addition, we identified different drug candidates for the same source when we considered gender and age, highlighting the importance of personalized medicine also in COVID-19. The mechanism of action (MOA), indications and clinical phases of the resulting drugs were further investigated to evaluate the potential of the candidates in the clinic (Table S2) (Wang et al., 2018).

## Discussion

Humanity has faced viral pandemics for years, which have led to health crises around the world. The major viral pandemics that have affected people worldwide in the last two decades include SARS-CoV-1 infection, influenza A H1N1 pandemic, Middle East Respiratory Syndrome-CoV infection, Ebola virus pandemic, and Zika virus epidemic (Bhadoria et al., 2021), and unfortunately, SARS-CoV-2 pandemic came into the scene in 2019. The ongoing COVID-19 pandemic has affected global health care and caused millions of deaths and cases worldwide. Therefore, these statistics clearly show us how serious the pandemics are and reveal the true extent of COVID-19.

In this study, we investigated the potential differences between the phenotypes of SARS-CoV-2 patients and healthy subjects from the perspective of personalized medicine, taking into account gender, age and origin of the samples. We highlighted potential differences between the two phenotypes at the mRNA level (i.e. DEG analysis), at the functional level (i.e. enrichment analysis) and at the therapeutic level (i.e. drug repurposing analysis). DEG analysis yielded tens, hundreds, or thousands of DEGs for each case. When we evaluated the number of case-specific and common core-DEGs between all conditions, we found that the number of case-specific core-DEGs was higher than that of common core-DEGs, indicating significant differences at the mRNA level depending on source, gender and age in COVID-19.

In addition, the core DEGs, which show statistically different expression at high and low viral load in different cases, generally consist of cytokines (*SOCS1*), chemokines, which are a family of small cytokines (*CXCL10, CXCL11* and *CXCL14*), and interferon-simulated genes (*IFIT1* and *IFIT2*), which the interferons comprising a family of cytokines. The expression of cytokines has been reported to correlate significantly with the severity of COVID-19 disease. Chemokines are crucial for an efficient host immune response, also in COVID-19 (Hsu et al., 2022). In addition, interferon molecules are the primary defence against viral infections and mediate protective mechanisms. IFITs prevent viral replication and these genes are silent by nature or expressed at low levels and induced after viral infection (Fensterl & Sen, 2015). A core-DEG *RUNX1*, which culminated in a young male case, is also a mediator of inflammatory signaling and is associated with an inflammatory response in lung diseases (Hu et al., 2022). Our analyses have therefore shown that the expression of cytokines (including chemokines and interferons) is significantly associated with viral load and is consistent with the results in the literature. However, as there are few core-DEGs associated with viral load expression, the significance of viral load response patterns between gender and age differences is not particularly clear.

To better understand the general signaling events in COVID-19 and potentially find the basis for targeted therapeutic developments against the infection, we performed enrichment analyzes. In support of the fact that any viral infection can trigger immune responses in the infected organism (Hosseini et al., 2020), we found signaling pathways involving interferon and cytokine signaling, highlighting the importance of innate and adaptive immune responses against COVID-19. Enrichment analysis of respiratory samples revealed signaling pathways generally related to immune response, while enrichment analysis of blood samples revealed pathways related to cell cycle processes in addition to those related to immunity. Viruses promote replication for their own benefit (i.e. to evade the host immune system) (Kori & Arga, 2020) and as with other viral infections, SARS-CoV-2 RNA in blood is a strong prognostic factor in the clinic. Considering the viruses' desire to replicate and the fact that blood serves as a source of viral replication, we expected to find cell cycle-related signaling pathways in the blood enrichment results. Furthermore, based on our enrichment results, we can predict that SARS-CoV-2 can be better controlled in the respiratory tract than in blood.

In this study, given the limitations of virus-directed drug therapies, we focused on the discovery of potential drug candidates against the host (i.e., host-directed drug candidates) (Cakir et al., 2021). These limitations include the lack of drugs targeting viral proteins and, more importantly, the rapid evaluation of the viral genome. Like all viruses, SARS-CoV-2 is susceptible to genetic changes to adapt to its hosts, and in SARS-CoV-2 we have encountered five significant variants (alpha, beta, gamma, delta, and omicron) (Ahmad et al., 2022) that differ greatly from the parent variant in their effects on humans. In addition, Remdesivir, the first FDA-approved antiviral drug for the treatment of COVID-19, is known to target viral RNA-dependent RNA polymerase (Malin et al., 2020), although the efficacy of the drug is still questioned by some authorities. Therefore, in order to represent biologically effective drugs and discover the host-oriented drug candidates, we integrated core-DEGs with SARS-CoV-2 and human PPI data.

A total of 63 drug candidates were identified by analyzing the repurposing of drugs, taking into account that the treatment of a disease may vary from patient to patient. This approach brings us to the concept of personalized medicine, which stands for the right drug for the right patient at the right time and at the right dose (Sadée & Dai, 2005). As expected, we obtained different drug candidates when we considered the sources of the samples as well as the gender and age of the individuals. We identified drug candidates that act as inhibitors or antagonists and are usually used to treat different types of cancer, such as leukemia, breast, lung, ovarian, prostate, pancreatic, renal cell and gastric cancer, etc. In addition to cancer treatment, some of the discovered drugs are also used to treat HIV-1 (nelfinavir), hypertension (reserpine), thrombosis (ticlopidine), fungi (cycloheximide), parasites (mebendazole, oxibendazole) and psychiatric disorders (SKF-77434, rimcazole and HU-211).

Evaluation of the discovered male-specific drug candidates shows that they generally target mechanisms related to the central nervous system, inflammation and infection, and cancer. On the other hand, most female-specific drugs block the cell cycle and induce apoptosis. A look at the age-specific culminated drugs also shows that drugs targeting cancer and infections are prominent in all age groups, while drugs affecting the central nervous system were only observed in the elderly group. Although 63 different drugs have been discovered, 15 of them are already being used in clinics today (Table S2). Of the drugs presented, the effects of albendazole and oxibendazole on COVID-19 were studied and found to have a protective effect against the disease (Panahi et al., 2022). Another introduced drug, mebendazole, has been associated with shorter hospital stays in COVID-19 patients (Galal et al., 2022). In addition, studies have shown that sirolimus (Singla et al., 2024) and ticlopidine (Tesei et al., 2022) show promising results in combating COVID-19. For the other approved drugs, there are no remarkable studies for COVID-19. Therefore, some of the drugs discovered in this study have already been efficiently linked to the disease in previous studies, which further strengthens our confidence in our observations and leads us to believe that the drug candidates reported here deserve further clinical efforts.

The main limitation of the study is the lack of experimental validation of the results with relevant tissue samples or cell lines. Therefore, in vitro or in vivo studies need to be conducted in the future to investigate and test the effects of the identified novel findings and drugs in terms of their response to the disease, cell viability, disease progression and migration. We believe that computational analysis is an important and first step in the development of biomarkers therapeutic targets and/or drugs. To address a broad medical audience, the need for experimental validation is therefore inevitable.

SARS CoV-2 is not the first pandemic humanity has faced, nor is it likely to be the last. We believe that such pandemics, which have affected large populations and significantly impacted public health, should be studied in depth from all perspectives, including the long-term effects. In this study, we investigated and highlighted the possible differences between the COVID-19 and control groups, taking into account the characteristics of the patients (i.e. gender and age differences) and the samples (i.e. blood and respiratory). Furthermore, using in silico analyses, we presented host-specific drug candidates that deserve to be validated by further experimental studies.

## Supplementary Information

Below is the link to the electronic supplementary material.

Supplementary file1 (PDF 198 KB)

## Data Availability

Publicly available datasets were analysed in this study. The datasets analysed during the current study are available in Gene Expression Omnibus (GEO) (https://www.ncbi.nlm.nih.gov/geo/).
