# Supplementary material for: Exploring COVID-19 Pandemic Disparities with Transcriptomic Meta-analysis from the Perspective of Personalized Medicine
Source: J Microbiol. 2024 Jul 9;62(9):785–98. doi: 10.1007/s12275-024-00154-9 (PMC11436439; doi:10.1007/s12275-024-00154-9)
Supplement: Supplementary file 1 — Supplementary file1 (PDF 198 KB) [file 12275_2024_154_MOESM1_ESM.pdf]

Table S1. The number of patients by viral load (i.e. high and viral load), the number of differentially expressed genes obtained, representing different expression patterns between the different viral conditions, and the resulting core-DEGs. Samples marked in red indicate that they could not be included in the analyses due to the inclusion criteria of the study.

CASE		GSE152075	GSE156063	GSE179277	GSE188678
**FEMALE**	***Number of samples***	152 high + 49 low	25 high + 25 low	27 high + 13 low	29 high + 17 low
	Number of DEGs	195	93	277	28
	Core-DEGs	CXCL10, CXCL11, CXCL14, IDO1, IFIT1, IFIT2, MT2A, PDGFA, SOCS1 and WARS1			
**MALE**	***Number of samples***	131 high + 45 low	19 high + 24 low	23 high + 20 low	22 high + 22 low
	Number of DEGs	656	4	81	133
	Core-DEGs	CLSPN, CXCL10 and CXCL11			
**FEMALE YOUNG**	***Number of samples***	44 high + 11 low	8 high + 11 low	9 high + 3 low	7 high + 6 low
	Number of DEGs	4	0	401	13
	Core-DEGs	None			
**FEMALE ADULT**	***Number of samples***	60 high + 15 low	12 high + 12 low	11 high + 9 low	9 high + 9 low
	Number of DEGs	46	143	37	10
	Core-DEGs	SLITRK2			
**FEMALE ELDER**	***Number of samples***	48 high + 23 low	5 high + 2 low	7 high + 1 low	6 high + 2 low
	Number of DEGs	363	3	244	19
	Core-DEGs	Not included in the analyses			
**MALE YOUNG**	***Number of samples***	30 high + 11 low	8 high + 6 low	12 high + 10 low	7 high + 5 low
	Number of DEGs	3619	59	777	15
	Core-DEGs	RUNX1T1			

Table S1. ***Continued.***

CASE		GSE152075	GSE156063	GSE179277	GSE188678
**MALE ADULT**	***Number of samples***	53 high + 23 low	8 high + 16 low	7 high + 9 low	5 high + 7 low
	Number of DEGs	158	22	38	35
	Core-DEGs	None			
**MALE ELDER**	***Number of samples***	48 high + 11 low	3 high + 2 low	4 high +1 low	6 high + 4 low
	Number of DEGs	63	9	38	252
	Core-DEGs	GRIA4			

Table S2. The drug candidates features that resulted according to the drug repurposing analysis (the drugs highlighted in blue represent common drug candidates for both sources)

Drug	Mechanism of action (MOA)	Indication	Phase
ABT-737	BCL inhibitor	-	Phase 1/Phase 2
ABT-751	Tubulin polymerization inhibitor	-	Phase 2
Albendazole	Tubulin polymerization inhibitor	Cystic hydatid disease, parenchymal neurocysticercosis	Launched
Amsacrine	Topoisomerase inhibitor	Acute lymphoblastic leukemia, acute lymphoblastic leukemia	Launched
AZD-7762	-	Anticancer drug	-
Baccatin-III	-	-	-
Berbamine	Calcium channel blocker	Advanced hepatocellular carcinoma	-
Blebbistatin	ATPase inhibitor	-	Preclinical
BRD-A09719808	-	-	-
BRD-K05402890	-	-	-
BRD-K37940862	-	-	-
BRD-K46681113	-	-	-
BRD-K57238941	-	-	-
BRD-K66902379	-	-	-
BRD-K69516039	-	-	-
BRD-K91047982	-	-	-
BRD-K96704748	-	-	-
Brivanib	FGFR inhibitor, VEGFR inhibitor	-	Phase 3
Chlorambucil	DNA inhibitor	Chronic lymphocytic leukemia, Hodgkin's lymphoma	Launched
CI-976	ACAT inhibitor	-	Phase 1
Cimetidine	Histamine receptor antagonist	Ulcer disease, gastroesophageal reflux disease, aggressive systemic mastocytosis, Zollinger-Ellison syndrome, endocrine adenoma	Launched
Cycloheximide	Protein synthesis inhibitor	-	Preclinical
Docetaxel	Tubulin polymerization inhibitor	Breast cancer, non-small cell lung cancer (NSCLC), prostate cancer, gastric adenocarcinoma, head and neck squamous cell carcinoma	Launched
EX-527	SIRT inhibitor	-	Phase 2
GSK-461364	PLK inhibitor	-	Phase 1
GW-843682X	PLK inhibitor	-	Preclinical
HU-211	NMDA receptor antagonist	Traumatic brain injuries and neurologic disorders	-

**Drug**	**Mechanism of action (MOA)**	**Indication**	**Phase**
Ispinesib	Kinesin inhibitor	Breast cancer, lung cancer, renal cell carcinoma, pediatric indications, ovarian cancer, head and neck cancer	Phase 2
Mebendazole	Tubulin polymerization inhibitor	Pinworm, whipworm, hookworm, ascariasis	Launched
Nelfinavir	HIV protease inhibitor	Human immunodeficiency virus (HIV-1)	Launched
Oxibendazole	Tubulin polymerization inhibitor	Strongyles	Launched
Perhexiline	Carnitine palmitoyltransferase inhibitor	Angina pectoris	Launched
PX-12	Thioredoxin inhibitor	Gastric cancer, and pancreatic cancer	Phase 2
QS-11	-	-	-
Reserpine	Vesicular monoamine transporter inhibitor	Hypertension	Launched
Rimcazole	Sigma receptor antagonist	-	Phase 1
Rucaparib	PARP inhibitor	Recurrent ovarian and prostate cancers	Phase 3
SA-63133	Casein kinase inhibitor	-	Preclinical
Salermide	Sirtuin inhibitor	Potential anticancer drug	-
SB-203580	p38 MAPK inhibitor	-	Preclinical
SB-218078	CHK inhibitor	Potential anticancer drug	Preclinical
SB-225002	CC chemokine receptor antagonist	-	Preclinical
Sirolimus	mTOR inhibitor	Organ rejection, lymphangioleiomyomatosis	Launched
SKF-77434	Dopamine receptor agonist	Potential treatment for cocaine addiction	Preclinical
SKF-96365	Calcium channel blocker	-	Preclinical
Sunitinib	PLK inhibitor	Renal cell carcinoma, gastrointestinal stromal tumor	Phase 1
SYK-inhibitor	Syk inhibitor	-	-
Terreic-acid	Bruton's tyrosine kinase (BTK) inhibitor	Potential antibacterial and anticancer drug	Preclinical
Ticlopidine	Purinergic receptor antagonist	Thrombosis, stroke	Launched
Tosedostat	Peptidase inhibitor	-	Phase 2
Tozasertib	Aurora kinase inhibitor	Potential anticancer drug	Phase 2
TWS-119	Glycogen synthase kinase inhibitor	-	Preclinical
Vinburnine	Adrenergic receptor antagonist	Stroke	Launched
Vincristine	Tubulin polymerization inhibitor	Acute lymphoblastic leukemia	Launched
Vinorelbine	Tubulin polymerization inhibitor	Non-small cell lung cancer	Launched

Drug	Mechanism of action (MOA)	Indication	Phase
VU-0365118-1	-	-	-
Wiskostatin	N-WASP inhibitor	-	-
YK-4279	Apoptosis inhibitor	Potential anticancer drug	Preclinical
ZK-164015	Estrogen receptor antagonist	-	Preclinical
ZM-336372	RAF inhibitor	-	Preclinical
